# Alteration of Cortical Volume and Thickness in Myalgic Encephalomyelitis/Chronic Fatigue Syndrome

**DOI:** 10.3389/fnins.2022.848730

**Published:** 2022-04-22

**Authors:** Kiran Thapaliya, Sonya Marshall-Gradisnik, Donald Staines, Jiasheng Su, Leighton Barnden

**Affiliations:** ^1^National Center for Neuroimmunology and Emerging Diseases, Menzies Health Institute Queensland, Griffith University, Gold Coast, QLD, Australia; ^2^Center for Advanced Imaging, The University of Queensland, Brisbane, QLD, Australia

**Keywords:** cortex, myalgic encephalomyelitis/chronic fatigue syndrome, International Consensus Criteria, sub-cortical regions, volume and thickness, clinical measures

## Abstract

Myalgic Encephalomyelitis/Chronic fatigue syndrome (ME/CFS) patients suffer from neurocognitive impairment. In this study, we investigated cortical volumetric and thickness changes in ME/CFS patients and healthy controls (HC). We estimated mean surface-based cortical volume and thickness from 18 ME/CFS patients who met International Consensus Criteria (ICC) and 26 HC using FreeSurfer. Vertex-wise analysis showed significant reductions in the caudal middle frontal gyrus (*p* = 0.0016) and precuneus (*p* = 0.013) thickness in ME/CFS patients compared with HC. Region based analysis of sub-cortical volumes found that amygdala volume (*p* = 0.002) was significantly higher in ME/CFS patients compared with HC. We also performed interaction-with-group regressions with clinical measures to test for cortical volume and thickness correlations in ME/CFS with opposite slopes to HC (abnormal). ME/CFS cortical volume and thickness regressions with fatigue, heart-rate variability, heart rate, sleep disturbance score, respiratory rate, and cognitive performance were abnormal. Our study demonstrated different cortical volume and thickness in ME/CFS patients and showed abnormal cortical volume and thickness regressions with key symptoms of ME/CFS patients.

## Introduction

Myalgic encephalomyelitis/chronic fatigue syndrome (ME/CFS) is a complex illness characterized by a range of symptoms that includes fatigue, malaise, headaches, sleep disturbances, difficulties with concentration, and cognitive function, and muscle pain (Baker and Shaw, 2007). The cognitive symptoms include deficits in memory, attention, reaction time, information processing speed, and free memory recall (Cockshell and Mathias, 2010). The severity of ME/CFS has been classified according to Fukuda criteria (Fukuda, 1994), Canadian Consensus Criteria (CCC) (Carruthers et al., 2003), and International Consensus Criteria (ICC) (Carruthers et al., 2011).

Brain magnetic resonance imaging (MRI) has been performed to study the pathophysiology of ME/CFS (Zeineh et al., 2014; Barnden et al., 2015, 2019; Kimura et al., 2019; Thapaliya et al., 2020). Analysis of early structural imaging was limited to qualitative radiologist report. White matter (WM) abnormalities were not more prevalent in ME/CFS compared to healthy controls (Greco et al., 1997). In contrast, white matter hyperintensity or sulcal or ventricular enlargement were more prevalent in ME/CFS patients than in healthy controls (21 vs. 2%) (Natelson et al., 1993). The more liberal classification of ME/CFS subjects in these studies confounds comparisons with more recent Fukuda, CCC or ICC studies. Thus a more recent study of CCC classified subjects using radiologist reporting found no differences (Barnden et al., 2011). Quantitative MRI found T1 weighted signal intensity in prefrontal white matter (indicative of myelination) increased with increasing ME/CFS severity (Barnden et al., 2015). More advanced MRI also reported increased T1 (myelin) levels in somatosensory WM, but decreased levels in the brainstem in ME/CFS (Natelson et al., 1993; Barnden et al., 2018). This was not detected in earlier T1 scans (Barnden et al., 2011) which emphasizes the advantage of more advanced MRI instrumentation (3T magnet with 64 channel head-neck coil vs. 1.5 T magnet with birdcage coil). The ratio of T1-weighted and T2-weighted images also showed higher signal intensity levels in white matter and basal ganglia regions (Thapaliya et al., 2020).

Voxel-based morphometry (VBM) based on high spatial resolution anatomical scans permits quantification of both regional and global volumes in individual subjects (Maksoud et al., 2020). Global gray and/or WM volume differences have been reported in ME/CFS in some studies (de Lange et al., 2005; Finkelmeyer et al., 2018), WM only (Zeineh et al., 2014) but not others (Zeineh et al., 2014; Shan et al., 2016; Barnden et al., 2018). Differences in regional gray and white matter volumes were also reported in ME/CFS patients (Okada et al., 2004; Puri et al., 2012; Finkelmeyer et al., 2018). Increased amygdala and insula volumes and decreased regional white matter volumes in the pons, midbrain, and right temporal lobe were reported in ME/CFS patients (Finkelmeyer et al., 2018). Reduced gray matter volume in the occipital lobes, the right angular gyrus and left parahippocampal gyrus was observed in ME/CFS patients (Puri et al., 2012). Smaller WM volumes for the left putamen, right caudate, and left cerebellum were also observed in female ME/CFS patients compared to control females (Addiego et al., 2021). A longitudinal study showed a significant decrease over 6 years of WM (arcuate fasciculus) volume in ME/CFS patients but not in healthy controls (Shan et al., 2016). A 3T MRI surface-based approach detected larger cortical thicknesses in five right hemisphere regions including two arcuate fasciculus end points in Fukuda ME/CFS (Zeineh et al., 2014).

Findings in ME/CFS of both positive and negative differences in global and regional gray and white matter volumes are therefore inconsistent (Shan et al., 2020). These inconsistent findings in ME/CFS motivated further investigation of volumetric and thickness differences in both cortical and sub-cortical regions using anatomical images from a 3T MRI scanner. The specific aims of this exploratory study were to test for cortical and sub-cortical volumetric and thickness differences in ME/CFS, and to explore interaction-with-group regressions between volume and thickness maps and clinical measures which test for opposite correlations in the two groups.

## Materials and Methods

### Participant Recruitment

The study was approved by the human ethics (HREC/15/QGC/63 and GU:2014/838) committee of Griffith University and the Gold Coast University Hospital where scanning was performed. Written informed consent was obtained from all individuals. 18 ME/CFS patients who met ICC criteria (Carruthers et al., 2011) and 26 age-matched healthy control subjects were recruited (see Table 1 for demographic information) through an online Lime survey. Furthermore, healthy controls and ME/CFS patients were excluded if they had an exclusionary medical disorder were: hyper/hypotensive, had an autoimmune dysfunction, attention deficit hyperactivity disorder, autoimmune disease, microvascular disease, or body mass index (BMI) > 35 or were pregnant or breastfeeding.

**TABLE 1:** Demographic and clinical characteristics of patients with ME/CFS and HC.

	ME/CFS (*n* = 18)	HC (*n* = 26)	*p*-value
Age	43.2 ± 10.7	43.1 ± 13.7	0.89
M/F	6/12	9/17	N/A
Fatigue	14.0 ± 18.5	71.7 ± 17.1	< 0.001
HRV (%)	27.3 ± 16.1	21.0 ± 8.7	0.19
HR	71.4 ± 10.9	65.47 ± 8.0	0.039
Resp	4.06 ± 1.2	4.0 ± 1.1	0.96,
SDS	7.0 ± 1.9	1.9 ± 1.5	< 0.001
Ment_all	34.86 ± 23.9	73.1 ± 0.7	< 0.001

*ME/CFS, Myalgic Encephalomyelitis/Chronic fatigue syndrome; M/F, Male/Female; HRV, Heart rate variability; HR, Heart rate; Resp, Respiration rate; SDS, SF36 Sleep disturbance score; Ment_all, SF36 mental score.*

### Clinical Measures

Clinical measures incorporated in cortical volume and thickness map regressions were collected as mentioned in Thapaliya et al. (2021). The 36-item SF36 short-form health survey questionnaire (Alonso et al., 1995), was completed by all subjects, and “Fatigue,” “SF36 physical (Phys_all)” and “SF36 mental scores (Ment_all)” were extracted. An “information processing score (Procinfo)” and a “Sleep disturbance score (SDS)” were obtained *via* a survey: “In the past month, how severe were the following symptoms (on a scale of 1–10, 1 being not a problem, 10 being extremely severe)” for symptoms “Difficulty processing information?” and “Sleep disturbances?” The “Heart rate (HR),” “Heart rate variability (HRV),” and “Respiratory rate (Resp)” were extracted from the power spectra of the pulse oximeter and respiration strap data recorded during a 15-min resting-state fMRI acquired in the same scanning session (“HR” and “Resp” from the frequency of the primary peak, and HRV from the full width at half maximum of the primary HR peak).

### Data acquisition

T1 weighted images for both ME/CFS and HC were acquired using a 3T Skyra MRI scanner (Siemens Healthcare, Erlangen, Germany) with a 64-channel head-neck coil (Nova Medical, Wilmington, NC, United States). Three-dimensional T1 weighted images were acquired using a T1 weighted magnetization prepared rapid gradient-echo (MPRAGE) sequence with a repetition time (TR) = 2,400 ms, echo time (TE) = 1.81 ms, flip-angle = 8°, acquisition matrix = 224 × 224 × 208, and voxel size 1 mm × 1 mm × 1 mm. The total acquisition time for T1w scans was 8:20 min:s.

### Image Analysis

FreeSurfer version 7.1.1 (Fischl, 2012) was run to generate cortical, sub-cortical volume and thickness from T1w images from ME/CFS patients and healthy controls using the Desikan Killiany parcelation scheme (Desikan et al., 2006). The default FreeSurfer command ‘‘recon-all’’ was run in a Macintosh computer (Operating system: Catalina, RAM = 36 GB, and core: 8). The ‘‘recon-all’’ processing includes motion correction, non-uniform intensity normalization, automated Talairach transformation, intensity normalization, removal of non-brain tissue, cortical parcelation, sub-cortical segmentation, gray and white matter boundary tessellation, automated topology correction, and surface deformation. Detailed information on the pipeline can be found here^1^. Skull stripping and gray and white matter boundaries were checked visually, and participants were excluded if segmentation showed any error. The recon-all was performed using the “qcache” option and the analysis were performed using volume and thickness data with 10 mm full-width half maximum separately on the left and right hemisphere.

### Statistical Analysis

We performed group comparison of left and right hemisphere using a general linear model (GLM) (Fischl, 2012) by computing vertex-by-vertex for analysis of cortical volume and thickness using FreeSurfer. Individual structural maps were combined into a single dataset and resampled into MNI space using the FreeSurfer command “mris_preproc” (Fischl, 2012). GLM analysis was performed on the concatenated data of the left and right hemispheres using the FreeSurfer command “mri_glmfit” (Fischl, 2012). The multiple comparisons correction (cluster correction) (Hagler et al., 2006) was performed using “mri_glmfit-sim” (Fischl, 2012) with setting vertex-wise threshold at 1.3 and cluster-wise p-threshold of 0.05 to control for false positives.

We also performed cortical volume and thickness interaction-with-group regressions with clinical parameters to test for different relationships in ME/CFS and HC groups, that is, an abnormal relationship in ME/CFS. To perform the group interaction, we used the FreeSurfer GLM method by creating a FreeSurfer Group Descriptor (FSGD) file that describes a group of subjects and their accompanying data^2^ and the contrast^3^. The design matrix is automatically created by FreeSurfer. The default method Different Offset Different Slopes (DODS) was used to perform group interaction in FreeSurfer. The “mri_glmfit” command was run with FSGD, and contrast and multiple comparison correction (cluster correction) was performed using FreeSurfer command line “mri_glmfit-sim.” The detail information about group interaction can be found in the given link https://surfer.nmr.mgh.harvard.edu/fswiki/FsTutorial/GroupAnalysis. The eight clinical parameters used as regressors were “HR,” “HRV,” “Phys_all,” “Procinfo,” “Ment_all,” “Resp,” and “SDS.” One ME/CFS patient was omitted from group interaction analysis due to missing clinical information (Procinfo, “Phys_all,” and “SDS”). ME/CFS patient data with clinical and autonomic measure outliers (one-“Procinfo,” one-“SDS,” and two-“Resp”) were also omitted from group interaction analysis.

Region-based statistical analysis was also performed on cortical and subcortical regions using SPSS version 27. All the statistical tests were controlled for age, gender, and total intracranial volume. Correction for multiple comparisons was implemented using false discovery rate (FDR).

## Results

### Group Comparison: Myalgic Encephalomyelitis/Chronic Fatigue Syndrome vs. Healthy Controls

**FIGURE 1:**
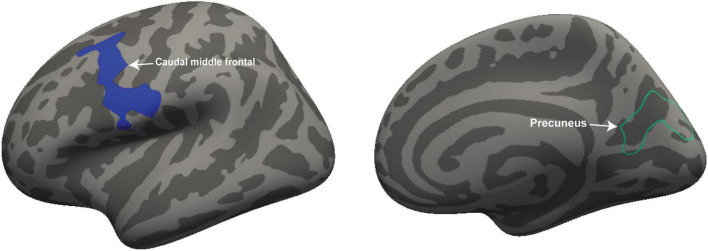
Volume and thickness reduction in ME/CFS patients. Volume was reduced in the left caudal middle frontal (white arrow) and thickness in the right precuneus region (white arrow) of ME/CFS patients compared with HC. The volume is represented with filled blue color whereas thickness is represented by unfilled green color. Significant volume and thickness clusters were overlaid on the inflated brain (left and right hemisphere) available in FreeSurfer.

We performed volumetric and thickness analysis on 18 ME/CFS patients and 26 HC. Figure 1 shows significant clusters with decreased volume in the left caudal middle frontal region (cluster size = 1,793 mm^2^, *p* = 0.0016, X = −34.6; Y = 2.6, Z = 53.8) and decreased thickness in the right precuneus region (cluster size = 1,418 mm^2^; *p* = 0.013; X = 23.1, Y = −63.1, Z = 12.4).

### Region-Based Analysis

We performed region-based analysis on the sub-cortical volume (Left and right: thalamus, caudate, putamen, pallidum, amygdala; posterior, anterior central regions of the corpus callosum; right, and total cortex volume) obtained directly from FreeSurfer as shown in Table 2. The central region of the corpus callosum, left and right hemisphere, and whole cortex volumes were significantly lower in ME/CFS compared to HC only before the multiple comparison correction (see Table 2). We only observed significantly *larger* volumes in left amygdala (*p* = 0.002) which survived the multiple comparison correction. The comparison of our significantly different volumetric regions in ME/CFS with previous findings are presented in Table 3.

**TABLE 2:** Vertex and region-based analysis of cortical regions in ME/CFS patients compared to HC.

Vertex based analysis				
	Areas	peak x y z (mm)	*p*	Cluster size
Volume	Left caudal middle frontal	−34 2 53	0.0016	1,793
Thickness	Right precuneus	23 −63 12	0.013	1,418

**Region based analysis**				

**Regions**	**ME/CFS**	**HC**	***p***	**95% confidence interval**

Left amygdala	1,758.5 ± 189.7	1,629.4 ± 130.2	0.002**	−234.7 to −59.1
CC central	536.2 ± 105.3	614.0 ± 134.6	0.014	20.6–172.4
Lh cortex	230,442.1 ± 20,425.5	245,579.6 ± 21,720.0	0.032	1,035.9–21,631.4
Rh cortex	230,753.3 ± 21,140.0	245,283.0 ± 21,343.8	0.041	478.1–21,429.5
Cortex	461,195.5 ± 41,542.0	490,862.7 ± 42,991.9	0.036	1,567.1–43,007.9

Vertex based analysis with reduced volume and thickness in ME/CFS. Sub-cortical regions with significantly higher/or lower volumes for ME/CFS than for HC, and p -values. Mean and standard deviation are represented as (±). CC, corpus callosum; Lh, left hemisphere; Rh, right hemisphere. Unit of volume is mm^3^. **Represents statistically significant after adjusting for multiple comparison.

**TABLE 3:** Different ME/CFS volumes reported here and in previous publications for both global and regional regions.

Author	Significantly different regions in ME/CFS compared to healthy controls		Sample size (ME/CFS)/HC	Diagnostic criteria
	Decreased	Increased		
This study	Volume: Left caudal middle frontal region Thickness: Right precuneus	Left amygdala	18/26	ICC
de Lange et al., 2005	Global Gray matter volume		13/15	Fukuda
Finkelmeyer et al., 2018	Global Gray matter volume Global White matter volume Bilateral internal and external capsule, anterior midbrain, pons, right prefrontal lone, inferior frontal lobe, anterior parts of the right temporal lobe	Right temporal lobe including insular cortex, bilateral amygdala, putamen, thalamus, parts of the left inferior frontal lobe and left occipital lobe	42/30	Fukuda
Okada et al., 2004	Bilateral prefrontal areas		16/49	Fukuda
Puri et al., 2012	Left and right occipital lobes (left lateral occipital cortex, superior division, and left supracalcrine cortex) Right angular gyrus and the left parahippocampal gyrus, posterior division White matter volume in the left occipital lobe		26/26	Fukuda
Zeineh et al., 2014	Supratentorial white matter volume	Right hemispheric cortical thickness (lateral occipital, precentral, middle temporal, post central and Pars orbitals	15/14	Fukuda
Addiego et al., 2021	Left putamen, right caudate and left cerebellum white matter		38/34	Fukuda and CCC
Shan et al., 2016	Left inferior fronto-occipital fasciculus		25/25	Fukuda and CCC

### Group Interaction: Myalgic Encephalomyelitis/Chronic Fatigue Syndrome vs. Healthy Controls

Vertex-based interaction-with-group regressions were performed between cortical volume and thickness (left and right hemisphere) surface maps and eight clinical scores: “Fatigue,” “Phys_all,” “Ment_all,” “Procinfo,” “SDS,” “HR,” “HRV,” and “Resp.” Significant volume and/or thickness interaction-with-group regressions were detected for six regressors (“Fatigue,” “HRV,” “HR,” “SDS,” “Resp,” “Ment_all”). Volume and thickness clusters for which ME/CFS regression slopes significantly different to HC slopes are listed in Table 4.

**TABLE 4:** Significant clusters from cortical volume and thickness voxel-wise interaction-with-group regressions with six clinical regressors.

Clinical parameter	Region		Cluster size mm^2^	MNI X Y Z mm	Cluster *p*
Fatigue (+)	Postcentral gyrus	RH/volume	3,570	38.3 −9.4 8.3	< 0.0001
	Inferior parietal lobe	RH/volume	1,625	44.7 −57 14.7	0.0028
	Inferior parietal lobe	RH/thickness	1,623	45.3 −51.4 41.5	0.0038
HRV (+)	Superior frontal gyrus	LH/thickness	1,920	−8.7 45.9 5.6	0.0024
HR (+)	Paracentral gyrus	LH/volume	1,920	−6.6 −32.2 58.7	0.0012
	Lateral occipital	LH/thickness	2,590	−34.8 −87.1 10	0.0002
	Lateral occipital	RH/thickness	2,203	30.5 −88.1 13.9	0.0002
	Caudal middle frontal	RH/thickness	1,384	41.7 16.9 47	0.015
SDS (+)	Lateral occipital	LH/volume	1,782	−43.8 −80.3 1.7	0.0016
	Superior frontal gyrus	LH/volume	1,731	−6.5 1 61.7	0.002
	Lingual gyrus	RH/thickness	1,302	12.2 −93.7 −8.4	0.02
Resp (-)	Caudal middle frontal	LH/volume	1,463	−37.1 0.6 33.6	0.009
	Superior frontal gyrus	RH/volume	1,213	16.4 −6.7 63.2	0.038
	Rostral middle frontal	LH/thickness	2,251	−36.7 19.2 22.4	0.0002
	Superior frontal gyrus	LH/thickness	1,325	−17.8 36.7 47.1	0.017
Ment_all (-)	Inferior parietal lobe	RH/volume	1,265	35.2 −79.6 20.2	0.028

*Clusters were formed with vertex-wise and cluster-wise p-thresholds of 0.05. The cluster p is corrected for multiple comparisons. The sign of the regressor is the sign of the slope of the regression for the ME/CFS group. LH, left hemisphere; RH, right hemisphere.*

**FIGURE 2:**
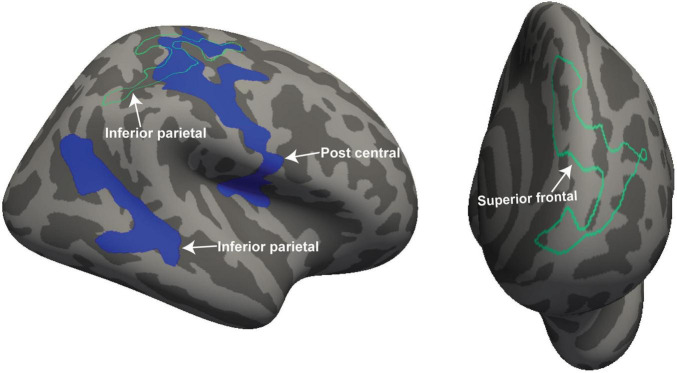
For ME/CFS and HC, significant clusters from interaction-with-group regressions for 2 clinical regressors (“Fatigue” and “HRV”). The volume and thickness cluster of the post central gyrus and inferior parietal was observed in the left hemisphere when regressed with “Fatigue” (left side). The thickness cluster of the superior frontal gyrus was detected at the left hemisphere when regressed with “HRV” (right side). The volume is represented with filled blue color whereas thickness is represented by unfilled green color. Significant volume and thickness clusters were overlaid on the inflated brain (left and right hemisphere) available in the FreeSurfer.

**FIGURE 3:**
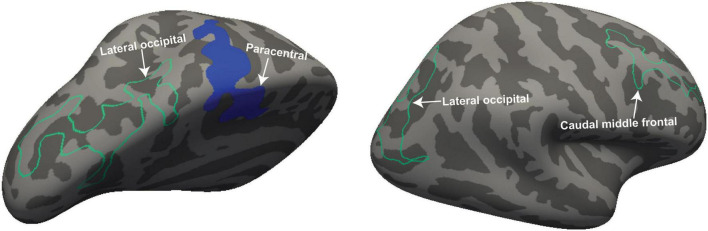
For ME/CFS and HC, a significant cluster from interaction-with-group regressions with “HR.” The volume cluster of the paracentral gyrus was observed in the left hemisphere and the thickness cluster of lateral occipital and caudal middle frontal gyrus in both left and right hemispheres. The volume is represented with filled blue color whereas thickness is represented by unfilled green color. Significant volume and thickness clusters were overlaid on the inflated brain (left and right hemisphere) available in the FreeSurfer.

**FIGURE 4:**
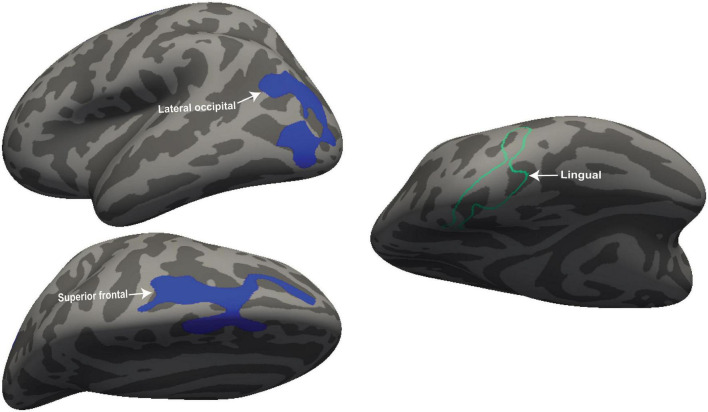
For ME/CFS and HC, a significant cluster from interaction-with-group regressions with “SDS.” The volume cluster of lateral occipital and superior frontal was observed in the left hemisphere and thickness cluster in the lingual gyrus in the right hemisphere. The volume (left hemisphere) is represented with filled blue color whereas thickness (right hemisphere) is represented by unfilled green color. Significant volume and thickness clusters were overlaid on the inflated brain (left and right hemisphere) available in the FreeSurfer.

**FIGURE 5:**
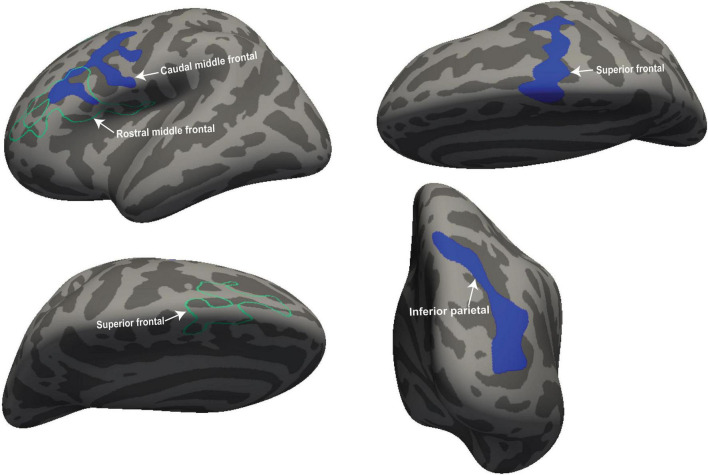
For ME/CFS and HC, a significant cluster from interaction-with-group regressions with “Resp” and “Ment_all.” The volume cluster of caudal middle frontal and superior frontal were observed in the left and right hemisphere and the thickness cluster of superior frontal when cortical volume and thickness regressed with “Resp.” The volume cluster of the inferior parietal lobe was detected at the right hemisphere when regressed with “Ment_all.” The volume is represented with filled blue color whereas thickness (right hemisphere) is represented by unfilled green color. Significant volume and thickness clusters were overlaid on the inflated brain (left and right hemisphere) available in the FreeSurfer.

**FIGURE 6:**
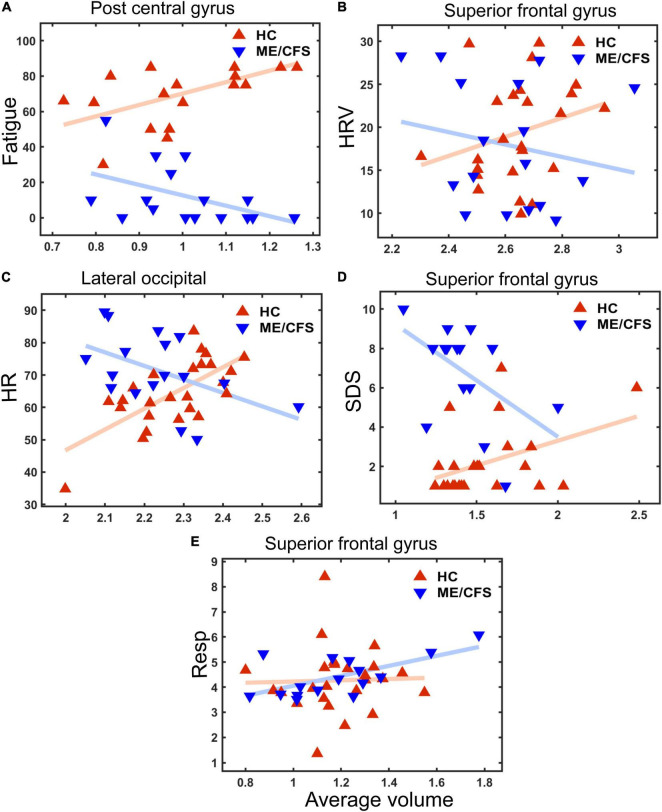
Plots for cluster average volume vs. five clinical measures (see Y-axis label). The X axis is “Average Volume,” the spatial average of the local volumes in the cluster. **(A)** Fatigue score (cluster *p* ≤ 0.0001 in postcentral gyrus—see Figure 2). **(B)** Heart Rate Variability (HRV) (cluster *P* = 0.0028 in superior frontal gyrus– see Figure 2); **(C)** Heart rate (HR) (cluster *p* = 0.0002 in lateral occipital– see Figure 3); **(D)** Sleep disturbance score (SDS) (*p* = 0.002 in superior frontal gyrus– see Figure 4); **(E)** Respiration Rate (Resp) (cluster *p* = 0.038 in superior frontal gyrus—see Figure 5). Lines are linear fits to individual values. Average volume (x-axis) was default volume obtained from the “mri_glmfit-sim” command from FreeSurfer that computes a spatial average inside a cluster.

Figure 2 shows four clusters with statistically significant volume or thickness interaction-with-group regressions with “Fatigue” and “HRV.” Fatigue showed a significantly different ME/CFS regressions in the right postcentral gyrus and inferior parietal lobe (see Figure 2, left). Cortical thickness interaction regressions with “HRV” showed significant clusters in the right superior parietal (Figure 2 left) and the left superior frontal gyrus (Figure 2 right). “HR” regressions showed significant clusters with abnormal volume and thickness in ME/CFS. The significant cluster of later occipitals (left and right) and caudal middle (right) frontal gyrus thickness and in the paracentral gyrus volume in the left hemisphere (see Figure 3). Cortical volume and thickness regression with “SDS” showed significant volume clusters in the left later occipital and superior frontal gyrus and significant thickness clusters in the right lingual gyrus (see Figure 4). Four significant volume and thickness clusters were detected in regression with “Resp” (see Figure 5). Significant volume cluster of left caudal middle frontal and right superior frontal gyrus and thickness cluster of left rostral middle frontal and superior frontal gyrus were abnormal in ME/CFS patients (see Figure 5). Cortical volume regression with “Ment_all” showed a significant cluster in the inferior parietal lobe of the right hemisphere (see Figure 5, right). The interaction-with group regression plot is shown in the Figure 6.

## Discussion

This study implemented surface-based analysis which defines internal and external cortex surfaces as a grid of vertices. At each vertex local cortical volume and thickness are computed. Here we performed vertex-by-vertex comparisons between the ME/CFS and HC groups for both volume and thickness. The advantage of the vertex-based approach is that it does not require any *a priori* hypothesis of locations of interest, unlike the region-based approach, and reports clusters of vertices. For display purposes the convoluted cortical gyrus maps are “inflated” to a smooth surface with shading to indicate original sulcal locations.

### Group Comparison: Myalgic Encephalomyelitis/Chronic Fatigue Syndrome vs. Healthy Controls

We detected significantly decreased volumes in the left caudal middle frontal cortex in ME/CFS patients. This region is involved in inhibition and modulation of attention (Japee et al., 2015) and participates in executive function (Andersson et al., 2009). A study of self-initiated elaborate encoding strategies (which rely on complex, highly effortful cognitive processes) demonstrated the involvement of left caudal middle frontal cortex (Husa et al., 2017). ME/CFS patients report memory and concentration problems, and difficulties in processing complex information (Jason et al., 1999) and perform worse than healthy controls in neuropsychological tests of attention, working memory, and processing speed (Marcel et al., 1996; Vercoulen et al., 1998). These deficits are consistent with the observed smaller left caudal middle frontal volume in ME/CFS.

Our ME/CFS patients also had reduced cortical thickness in the right precuneus which is involved in visual imagery, attention, and memory retrieval (Cavanna and Trimble, 2006). This is consistent with the ME/CFS symptom of difficulty in directing and maintaining visual attention (Hutchinson and Badham, 2013).

We also detected significant differences in the left amygdala volume in ME/CFS patients. The volume of the amygdala was significantly greater in ME/CFS which confirms an earlier VBM result (Finkelmeyer et al., 2018). Amygdala morphological changes can indicate a neuroinflammatory process (Lv et al., 2014; Nakatomi et al., 2014) or neuronal and synaptic alterations induced by stress (Roozendaal et al., 2009; Christoffel et al., 2011). Increased financial stress was associated with increased symptom severity in ME/CFS (Balinas et al., 2021) and better stress management skills lowered illness burden and fatigue severity in ME/CFS (Lattie et al., 2013). Increased amygdala volume in ME/CFS from exposure to stress may be mediated by the expression of Brain-derived neurotrophic factor (BDNF) (Bennett and Lagopoulos, 2014) which is altered in ME/CFS (Chen et al., 2008; Polli et al., 2020).

### Group Interaction: Myalgic Encephalomyelitis/Chronic Fatigue Syndrome vs. Healthy Controls

Vertex-based cortical volume and thickness interaction-with-group regressions with clinical measures yielded multiple significant clusters (Table 4 and Figures 2–5). In these clusters, regressions were oppositely directed for ME/CFS and HC, that is, ME/CFS regressions were abnormal (see Figure 6). We interpret inter-individual differences in local volume or thickness to be an expression of normal human variability. Figure 6 (x-values) shows this is similar for both ME/CFS and HC in the clusters illustrated. Insofar as volume or thickness is a surrogate for a functionally relevant feature such as myelination or axonal density, different correlations with clinical measures in a cluster indicate abnormal communication in ME/CFS within the control circuits that traverse the cluster and influence the clinical measure. This mechanism was proposed in an earlier MRI study of autonomic correlations (Barnden et al., 2016).

Cortical volume and thickness map interaction-with-group regressions with “Fatigue” and “Ment_all” scores both showed significant clusters in the inferior parietal lobe. The inferior parietal lobe is a hub of the default mode network (DMN) and the abnormal correlations detected here with fatigue and mental scores may be a manifestation of the same neuronal phenomenon that yielded diminished resting connectivity between inferior parietal and medial prefrontal DMN hubs in the same cohort (Shan et al., 2018).

We detected significant cortical thickness interaction regression with heart rate variability (HRV) in the left superior frontal gyrus. This is consistent with a resting-state functional MRI study which showed that HRV was positively correlated with BOLD activity in the superior frontal gyrus (Yoo et al., 2018).

We also demonstrated an abnormal correlation between respiratory rate (Resp) and cortical volume and thickness in the superior frontal gyrus, caudal middle frontal, and rostral middle frontal cortex. A pilot study in ME/CFS showed different respiratory rates in ME/CFS patients (Nijs et al., 2008). Our previous T1/T2 study also showed a group interaction with respiratory rate in the middle temporal gyrus, corpus callosum, and cerebral WM regions in ME/CFS patients (Thapaliya et al., 2020). A diffusion tensor imaging (DTI) study found an abnormal correlation between diffusion parameters correlation and “Resp” (Thapaliya et al., 2021) in the superior prefrontal cortex (BA 9) in ME/CFS patients.

Here we also detected abnormal cortical volume and thickness interaction-with-group regressions with “HR” in four cortical regions (Table 4), one in the middle frontal lobe. HR is faster in ME/CFS than controls in both supine and seated positions (Nelson et al., 2019). White matter (WM) volumes from voxel-based morphometry showed interaction-with-group regressions in bilateral prefrontal WM, hypothalamus and cerebellum (Barnden et al., 2016).

The autonomic measures HRV, Resp, and HR are regulated by the central autonomic network that involves the medial prefrontal cortex, insular cortex, amygdala, hypothalamus and midbrain, pons and medulla (Benarroch, 1993). Here the prefrontal cortex was involved in multiple interaction with group regressions with autonomic measures.

We also tested cortical volume and thickness maps for interaction-with group regressions with sleep disturbance score (SDS). Significant clusters were detected in the superior frontal, lingual and occipital cortex. Previous research on alcohol use disorder patients with sleep disorder showed reduced overall cortical volume (Wiers et al., 2015; Tomasi et al., 2019). Zhang et al. (2021) showed that longer sleep-wave and rapid eye movement (REM) sleep was significantly associated with greater cortical thickness. Diffusion tensor imaging showed abnormal inferior frontal gyrus correlations between “SDS” and DTI parameters in ME/CFS patients (Thapaliya et al., 2021). Another study using fMRI also showed activation of the inferior frontal gyrus after sleep deprivation (Vartanian et al., 2014). Thus, the clusters detected here do not agree with earlier “SDS” results and further study is required to resolve this difference.

### Limitations

The relatively small ME/CFS sample size will affect the power of the study to detect all the differences in cortical regions and their association with clinical measures. Larger populations should be investigated in future studies to ensure more accurate statistical results are obtained. The cortical volume and thickness are also affected by the choice of work station, operating system, processing software, and its version (Gronenschild et al., 2012; Perlaki et al., 2017; Seiger et al., 2018). Another limitation is that some of the clinical scores in this study were obtained by questionnaires, which by their subjective nature may limit interpretation of our findings. This study was a cross-sectional study. Longitudinal studies should be performed to test for progressive cortical volume and thickness changes in ME/CFS patients.

## Conclusion

Our study detected significantly reduced cortical volume and thickness in ME/CFS patients compared with HC. We found that amygdala volume was significantly higher in ME/CFS patients. We also observed that cortical volume and thickness relationships were abnormal in regressions with clinical and autonomic measures. Overall, our findings suggest altered cortical volume and thickness in ME/CFS patients relative to healthy controls.

## Data Availability Statement

The original contributions presented in the study are included in the article/supplementary material, further inquiries can be directed to the corresponding author/s.

## Ethics Statement

The studies involving human participants were reviewed and approved by HREC/15/QGC/63 and GU:2014/838. The patients/participants provided their written informed consent to participate in this study.

## Author Contributions

KT: project design, data analysis, methodology, writing-original draft, and writing-review and editing. LB: supervision, methodology, and writing-review and editing. DS: supervision and writing—review and editing. SM-G: supervision and writing—review and editing. JS: writing-review and editing. All authors contributed to the article and approved the submitted version.

## Conflict of Interest

The authors declare that the research was conducted in the absence of any commercial or financial relationships that could be construed as a potential conflict of interest.

## Publisher’s Note

All claims expressed in this article are solely those of the authors and do not necessarily represent those of their affiliated organizations, or those of the publisher, the editors and the reviewers. Any product that may be evaluated in this article, or claim that may be made by its manufacturer, is not guaranteed or endorsed by the publisher.

## Footnotes

1
https://surfer.nmr.mgh.harvard.edu/fswiki/recon-all

2
https://surfer.nmr.mgh.harvard.edu/fswiki/FsgdFormat

3
https://surfer.nmr.mgh.harvard.edu/fswiki/Fsgdf2G2V

## References

[B1] Addiego F. M. Zajur K. Knack S. Jamieson J. Rayhan R. U. Baraniuk J. N. (2021). Subcortical brain segment volumes in gulf war illness and myalgic encephalomyelitis/chronic fatigue syndrome. *Life Sci.* 282:119749. 10.1016/j.lfs.2021.119749 34214570

[B2] Alonso J. Prieto L. Anto J. M. (1995). The Spanish version of the SF-36 Health Survey (the SF-36 health questionnaire): an instrument for measuring clinical results. *Med. Clín.* 104 771–776. 7783470

[B3] Andersson M. Ystad M. Lundervold A. Lundervold A. J. (2009). Correlations between measures of executive attention and cortical thickness of left posterior middle frontal gyrus - a dichotic listening study. *Behav. Brain Funct.* 5:41. 10.1186/1744-9081-5-41 19796388 PMC2761925

[B4] Baker R. Shaw E. J. (2007). Diagnosis and management of chronic fatigue syndrome or myalgic encephalomyelitis (or encephalopathy): summary of NICE guidance. *BMJ* 335 446–448. 10.1136/bmj.39302.509005.AE 17762037 PMC1962830

[B5] Balinas C. Eaton-Fitch N. Maksoud R. Staines D. Marshall-Gradisnik S. (2021). Impact of life stressors on myalgic encephalomyelitis/chronic fatigue syndrome symptoms: an australian longitudinal study. *Int. J. Environ. Res. Public Health* 18:10614. 10.3390/ijerph182010614 34682360 PMC8535742

[B6] Barnden L. R. Crouch B. Kwiatek R. Burnet R. Del Fante P. (2015). Evidence in chronic fatigue syndrome for severity-dependent upregulation of prefrontal myelination that is independent of anxiety and depression. *NMR Biomed.* 28 404–413. 10.1002/nbm.3261 25702943 PMC4369127

[B7] Barnden L. R. Crouch B. Kwiatek R. Burnet R. Mernone A. Chryssidis S. (2011). A brain MRI study of chronic fatigue syndrome: evidence of brainstem dysfunction and altered homeostasis. *NMR Biomed.* 24 1302–1312. 10.1002/nbm.1692 21560176 PMC4369126

[B8] Barnden L. R. Kwiatek R. Crouch B. Burnet R. Del Fante P. (2016). Autonomic correlations with MRI are abnormal in the brainstem vasomotor centre in chronic fatigue syndrome. *NeuroImage Clin.* 11 530–537. 10.1016/j.nicl.2016.03.017 27114901 PMC4833047

[B9] Barnden L. R. Shan Z. Y. Staines D. R. Marshall-Gradisnik S. Finegan K. Ireland T. (2018). Hyperintense sensorimotor T1 spin echo MRI is associated with brainstem abnormality in chronic fatigue syndrome. *Neuroimage Clin.* 20 102–109. 10.1016/j.nicl.2018.07.011 30497131 PMC6309570

[B10] Barnden L. R. Shan Z. Y. Staines D. R. Marshall-Gradisnik S. Finegan K. Ireland T. (2019). Intra brainstem connectivity is impaired in chronic fatigue syndrome. *NeuroImage Clin.* 24:102045. 10.1016/j.nicl.2019.102045 31671321 PMC6835065

[B11] Benarroch E. E. (1993). The central autonomic network: functional organization, dysfunction, and perspective. *Mayo Clin. Proc.* 68 988–1001. 10.1016/s0025-6196(12)62272-1 8412366

[B12] Bennett M. R. Lagopoulos J. (2014). Stress and trauma: BDNF control of dendritic-spine formation and regression. *Prog. Neurobiol.* 112 80–99. 10.1016/j.pneurobio.2013.10.005 24211850

[B13] Carruthers B. M. Jain A. K. De Meirleir K. L. Peterson D. L. Klimas N. G. Lerner A. M. (2003). Myalgic encephalomyelitis/chronic fatigue syndrome: clinical working case definition, diagnostic and treatment protocols. *J. Chronic Fatigue Syndrome* 11 7–115. 10.1300/j092v11n01_02

[B14] Carruthers B. M. van de Sande M. I. Meirleir K. L. D. Klimas N. G. Broderick G. Mitchell T. (2011). Myalgic encephalomyelitis: International Consensus Criteria. *J. Int. Med.* 270 327–338. 10.1111/j.1365-2796.2011.02428.x PMC3427890 21777306

[B15] Cavanna A. E. Trimble M. R. (2006). The precuneus: a review of its functional anatomy and behavioural correlates. *Brain* 129 564–583. 10.1093/brain/awl004 16399806

[B16] Chen R. Liang F. X. Moriya J. Yamakawa J. Sumino H. Kanda T. (2008). Chronic fatigue syndrome and the central nervous system. *J. Int. Med. Res.* 36 867–874. 18831878 10.1177/147323000803600501

[B17] Christoffel D. J. Golden S. A. Russo S. J. (2011). Structural and synaptic plasticity in stress-related disorders. *Rev. Neurosci.* 22 535–549. 10.1515/RNS.2011.044 21967517 PMC3212803

[B18] Cockshell S. J. Mathias J. L. (2010). Cognitive functioning in chronic fatigue syndrome: a meta-analysis. *Psychol. Med.* 40 1253–1267. 10.1017/S0033291709992054 20047703

[B19] de Lange F. P. Kalkman J. S. Bleijenberg G. Hagoort P. van der Meer J. W. M. Toni I. (2005). Gray matter volume reduction in the chronic fatigue syndrome. *Neuroimage* 26 777–781. 10.1016/j.neuroimage.2005.02.037 15955487

[B20] Desikan R. S. Ségonne F. Fischl B. Quinn B. T. Dickerson B. C. Blacker D. (2006). An automated labeling system for subdividing the human cerebral cortex on MRI scans into gyral based regions of interest. *Neuroimage* 31 968–980. 10.1016/j.neuroimage.2006.01.021 16530430

[B21] Finkelmeyer A. He J. Maclachlan L. Watson S. Gallagher P. Newton J. L. (2018). Grey and white matter differences in chronic fatigue syndrome - a voxel-based morphometry study. *Neuroimage Clin.* 17 24–30. 10.1016/j.nicl.2017.09.024 29021956 PMC5633338

[B22] Fischl B. (2012). FreeSurfer. *NeuroImage* 62 774–781. 10.1016/j.neuroimage.2012.01.021 22248573 PMC3685476

[B23] Fukuda K. (1994). The Chronic Fatigue Syndrome: A Comprehensive Approach to Its Definition and Study. *Ann. Intern. Med.* 121:953. 10.7326/0003-4819-121-12-199412150-00009 7978722

[B24] Greco A. Tannock C. Brostoff J. Costa D. C. (1997). Brain MR in chronic fatigue syndrome. *AJNR Am. J. Neuroradiol.* 18 1265–1269. 9282853 PMC8338019

[B25] Gronenschild E. H. B. M. Habets P. Jacobs H. I. L. Mengelers R. Rozendaal N. van os J. (2012). The effects of freesurfer version, workstation type, and macintosh operating system version on anatomical volume and cortical thickness measurements. *PLoS One* 7:e38234. 10.1371/journal.pone.0038234 22675527 PMC3365894

[B26] Hagler D. J. Saygin A. P. Sereno M. I. (2006). Smoothing and cluster thresholding for cortical surface-based group analysis of fMRI data. *NeuroImage* 33 1093–1103. 10.1016/j.neuroimage.2006.07.036 17011792 PMC1785301

[B27] Husa R. A. Gordon B. A. Cochran M. M. Bertolin M. Bond D. N. Kirchhoff B. A. (2017). Left caudal middle frontal gray matter volume mediates the effect of age on self-initiated elaborative encoding strategies. *Neuropsychologia* 106 341–349. 10.1016/j.neuropsychologia.2017.10.004 28987907 PMC5908722

[B28] Hutchinson C. V. Badham S. P. (2013). Patterns of abnormal visual attention in myalgic encephalomyelitis. *Optom. Vis. Sci.* 90 607–614. 10.1097/OPX.0b013e318294c232 23689679

[B29] Japee S. Holiday K. Satyshur M. D. Mukai I. Ungerleider L. G. (2015). A role of right middle frontal gyrus in reorienting of attention: a case study. *Front. Syst. Neurosci.* 9:23. 10.3389/fnsys.2015.00023 25784862 PMC4347607

[B30] Jason L. A. Richman J. A. Rademaker A. W. Jordan K. M. Plioplys A. V. Taylor R. R. (1999). A community-based study of chronic fatigue syndrome. *Arch. Intern Med.* 159 2129–2137. 10527290 10.1001/archinte.159.18.2129

[B31] Kimura Y. Sato N. Ota M. Shigemoto Y. Morimoto E. Enokizono M. (2019). Brain abnormalities in myalgic encephalomyelitis/chronic fatigue syndrome: evaluation by diffusional kurtosis imaging and neurite orientation dispersion and density imaging. *J. Magn. Reson. Imaging* 49 818–824. 10.1002/jmri.26247 30430664

[B32] Lattie E. G. Antoni M. H. Fletcher M. A. Czaja S. Perdomo D. Sala A. (2013). Beyond myalgic encephalomyelitis/chronic fatigue syndrome (ME/CFS) symptom severity: stress management skills are related to lower illness burden. *Fatigue* 1:10. 10.1080/21641846.2013.843255 PMC3837381 24278791

[B33] Lv R.-J. Sun Z.-R. Cui T. Guan H.-Z. Ren H.-T. Shao X.-Q. (2014). Temporal lobe epilepsy with amygdala enlargement: a subtype of temporal lobe epilepsy. *BMC Neurol.* 14:194. 10.1186/s12883-014-0194-z 25269594 PMC4210593

[B34] Maksoud R. du Preez S. Eaton-Fitch N. Thapaliya K. Barnden L. Cabanas H. (2020). A systematic review of neurological impairments in myalgic encephalomyelitis/chronic fatigue syndrome using neuroimaging techniques. *PLoS One* 15:e0232475. 10.1371/journal.pone.0232475 32353033 PMC7192498

[B35] Marcel B. Komaroff A. L. Fagioli L. R. Kornish R. J. Albert M. S. (1996). Cognitive deficits in patients with chronic fatigue syndrome. *Biol. Psychiatry* 40 535–541. 10.1016/0006-3223(95)00422-x 8879474

[B36] Nakatomi Y. Mizuno K. Ishii A. Wada Y. Tanaka M. Tazawa S. (2014). Neuroinflammation in patients with chronic fatigue syndrome/myalgic encephalomyelitis: An 11C-(R)-PK11195 PET study. *J. Nucl. Med.* 55 945–950. 10.2967/jnumed.113.131045 24665088

[B37] Natelson B. H. Cohen J. M. Brassloff I. Lee H.-J. (1993). A controlled study of brain magnetic resonance imaging in patients with the chronic fatigue syndrome. *J. Neurol. Sci.* 120 213–217. 10.1016/0022-510x(93)90276-5 8138812

[B38] Nelson M. J. Bahl J. S. Buckley J. D. Thomson R. L. Davison K. (2019). Evidence of altered cardiac autonomic regulation in myalgic encephalomyelitis/chronic fatigue syndrome. *Med. (Baltimore)* 98:e17600. 10.1097/MD.0000000000017600 31651868 PMC6824690

[B39] Nijs J. Adriaens J. Schuermans D. Buyl R. Vincken W. (2008). Breathing retraining in patients with chronic fatigue syndrome: a pilot study. *Physiother. Theory Pract.* 24 83–94. 10.1080/09593980701429406 18432511

[B40] Okada T. Tanaka M. Kuratsune H. Watanabe Y. Sadato N. (2004). Mechanisms underlying fatigue: a voxel-based morphometric study of chronic fatigue syndrome. *BMC Neurol.* 4:14. 10.1186/1471-2377-4-14 15461817 PMC524491

[B41] Perlaki G. Horvath R. Nagy S. A. Bogner P. Doczi T. Janszky J. (2017). Comparison of accuracy between FSL’s FIRST and Freesurfer for caudate nucleus and putamen segmentation. *Sci. Rep.* 7:2418. 10.1038/s41598-017-02584-5 28546533 PMC5445091

[B42] Polli A. Ghosh M. Bakusic J. Ickmans K. Monteyne D. Velkeniers B. (2020). DNA methylation and brain-derived neurotrophic factor expression account for symptoms and widespread hyperalgesia in patients with chronic fatigue syndrome and comorbid fibromyalgia. *Arthritis Rheumatol.* 72 1936–1944. 10.1002/art.41405 32562379

[B43] Puri B. K. Jakeman P. M. Agour M. Gunatilake K. D. R. Fernando K. A. C. Gurusinghe A. I. (2012). Regional grey and white matter volumetric changes in myalgic encephalomyelitis (chronic fatigue syndrome): a voxel-based morphometry 3 T MRI study. *Br. J. Radiol.* 85 e270–e273. 10.1259/bjr/93889091 22128128 PMC3474083

[B44] Roozendaal B. McEwen B. S. Chattarji S. (2009). Stress, memory and the amygdala. *Nat. Rev. Neurosci.* 10 423–433. 10.1038/nrn2651 19469026

[B45] Seiger R. Ganger S. Kranz G. S. Hahn A. Lanzenberger R. (2018). Cortical thickness estimations of freesurfer and the CAT12 toolbox in patients with Alzheimer’s Disease and healthy controls. *J. Neuroimaging* 28 515–523. 10.1111/jon.12521 29766613 PMC6174993

[B46] Shan Z. Y. Barnden L. R. Kwiatek R. A. Bhuta S. Hermens D. F. Lagopoulos J. (2020). Neuroimaging characteristics of myalgic encephalomyelitis/chronic fatigue syndrome (ME/CFS): a systematic review. *J. Trans. Med.* 18:335. 10.1186/s12967-020-02506-6 32873297 PMC7466519

[B47] Shan Z. Y. Finegan K. Bhuta S. Ireland T. Staines D. R. Marshall-Gradisnik S. M. (2018). Decreased connectivity and increased blood oxygenation level dependent complexity in the default mode network in individuals with chronic fatigue syndrome. *Brain Connect.* 8 33–39. 10.1089/brain.2017.0549 29152994

[B48] Shan Z. Y. Kwiatek R. Burnet R. Del Fante P. Staines D. R. Marshall-Gradisnik S. M. (2016). Progressive brain changes in patients with chronic fatigue syndrome: a longitudinal MRI study. *J. Magn. Reson. Imaging* 44 1301–1311. 10.1002/jmri.25283 27123773 PMC5111735

[B49] Thapaliya K. Marshall-Gradisnik S. Staines D. Barnden L. (2020). Mapping of pathological change in chronic fatigue syndrome using the ratio of T1- and T2-weighted MRI scans. *NeuroImage: Clin.* 28:102366. 10.1016/j.nicl.2020.102366 32777701 PMC7417892

[B50] Thapaliya K. Marshall-Gradisnik S. Staines D. Barnden L. (2021). Diffusion tensor imaging reveals neuronal microstructural changes in myalgic encephalomyelitis/chronic fatigue syndrome. *Eur. J. Neurosci.* 54 6214–6228. 10.1111/ejn.15413 34355438 PMC9291819

[B51] Tomasi D. G. Wiers C. E. Shokri-Kojori E. Zehra A. Ramirez V. Freeman C. (2019). Association between reduced brain glucose metabolism and cortical thickness in alcoholics: evidence of neurotoxicity. *Int. J. Neuropsychopharmacol.* 22 548–559. 10.1093/ijnp/pyz036 31369670 PMC6754735

[B52] Vartanian O. Bouak F. Caldwell J. L. Cheung B. Cupchik G. Jobidon M.-E. (2014). The effects of a single night of sleep deprivation on fluency and prefrontal cortex function during divergent thinking. *Front. Hum. Neurosci.* 8:214. 10.3389/fnhum.2014.00214 24795594 PMC4001002

[B53] Vercoulen J. H. Bazelmans E. Swanink C. M. Galama J. M. Fennis J. F. van der Meer J. W. (1998). Evaluating neuropsychological impairment in chronic fatigue syndrome. *J. Clin. Exp. Neuropsychol.* 20 144–156. 10.1076/jcen.20.2.144.1160 9777468

[B54] Wiers C. E. Gawron C. K. Gröpper S. Spengler S. Stuke H. Lindenmeyer J. (2015). Decreased gray matter volume in inferior frontal gyrus is related to stop-signal task performance in alcohol-dependent patients. *Psychiatry Res. Neuroimaging* 233 125–130. 10.1016/j.pscychresns.2015.05.006 26078198

[B55] Yoo H. J. Thayer J. F. Greening S. Lee T.-H. Ponzio A. Min J. (2018). Brain structural concomitants of resting state heart rate variability in the young and old: evidence from two independent samples. *Brain Struct. Funct.* 223 727–737. 10.1007/s00429-017-1519-7 28921167 PMC5828882

[B56] Zeineh M. M. Kang J. Atlas S. W. Raman M. M. Reiss A. L. Norris J. L. (2014). Right arcuate fasciculus abnormality in chronic fatigue syndrome. *Radiology* 274 517–526. 10.1148/radiol.14141079 25353054

[B57] Zhang R. Tomasi D. Manza P. Shokri-Kojori E. Demiral S. B. Feldman D. E. (2021). Sleep disturbances are associated with cortical and subcortical atrophy in alcohol use disorder. *Transl. Psychiatry* 11 1–11. 10.1038/s41398-021-01534-0 34400604 PMC8368207

